# Effect of Practice of Physical Activity and Satisfaction with Health Status on Older Adults’ Perceived Motor Competence

**DOI:** 10.3390/ijerph22040606

**Published:** 2025-04-11

**Authors:** José C. Calero-Cano, María Espada, Javier Abián-Vicén, Pablo Abián

**Affiliations:** 1 Grupo de Investigación en Ciencias de la Actividad Física y Deporte (GICAF), Faculty of Humanities and Social Sciences, Comillas Pontifical University, 28108 Madrid, Spain; jccalero@comillas.edu; 2 Faculty of Health Sciences, King Juan Carlos University, 28922 Alcorcón, Spain; 3 Performance and Sport Rehabilitation Laboratory (DeporSalud), Faculty of Sports Sciences, University of Castilla-La Mancha, 45071 Toledo, Spain; javier.abian@uclm.es; 4 Department of Biomedical Sciences, Faculty of Medicine and Health Sciences, University of Alcala, 28801 Alcalá de Henares, Spain; pablo.abian@uah.es

**Keywords:** older adults, motor competence, physical activity, self-perceived health

## Abstract

Due to the increase in the number of elderly people in the world, research related to the elderly is becoming more and more relevant, especially with the objective of understanding the factors which can affect the preservation of an adequate quality of life. The objectives of this research were to analyze older adults’ perceived motor competence according to the practice of physical activity, satisfaction with health status, and social and demographic characteristics. This research followed a descriptive quantitative methodology using a survey. The “Standardized Questionnaire on Physical Activity and the Elderly” was administered to a total of 933 Spanish older adults (≥65 years old) (46.9% men, 53.1% women). The results show that the people who engaged in physical activity and/or sport on a regular basis had the best perception of their motor competence (*p* < 0.001). The more satisfied the subjects were with their health status, the higher the scores they obtained in their perceived motor competence (*p* < 0.001). Finally, the younger age group (≤74 years old), men, and people of a higher social class perceived their motor competence more satisfactorily (*p* < 0.001). The significance level was set at *p* < 0.05. The conclusions indicate a positive effect of physical activity on the perception of motor competence in older adults.

## 1. Introduction

In the current international context, research related to older adults is gaining more and more relevance. This is due, on the one hand, to the existence of an inverted population pyramid worldwide, due to the low birthrate and the increase in the average age of mortality, and on the other, to increasing social awareness of aging with adequate functional independence and, as a result, conserving an adequate quality of life. Population aging is a phenomenon referred to by some authors as a “silent revolution”, something which is more accentuated in western societies.

At the global level, the population of people older than 60 was 960 million in 2017. It is expected that, in 2050, the number of people in this age group will have doubled, reaching nearly 2.1 billion [1]. In Spain, in 2024, the population of people over 64 represented 20.42% of the total, an increase of about 3% in the last decade [2].

When dealing with the quality of life of older adults in relation to their health status, not only physical or biological aspects should be taken into account but also all the dimensions of the person (psychological, social, affective, etc.), as stated by Schwartzmann [3]. Furthermore, awareness of one’s own health status also indicates the general state of aging. This same author points to the need to add the subjective perception of the patients to research procedures, as bearing it in mind is a reliable way to contribute empirical tests with a proven scientific basis to the decision-making process in health matters. In this same vein, Whaley and Schrider [4] explain that understanding self-perceptions can help in the designing of effective interventions that foster the participation and commitment of older adults to physical activity programs.

In this regard, research like that of Ruthig et al. [5] reveals that a good perception of one’s own health status can be a good indicator of one’s own different health risks. In this research, the health status of the older adults was estimated as 3.59 ± 0.68 on a range of 2 to 5, with 2 being a very bad status and 5 being very good.

Moreover, the decrease in functional capacity and the appearance of health problems provoke a reduction in physical activity, which, added to social and personal circumstances, constitutes a risk factor in the older population [6].

Chronological age is a handy, and often very good, predictor of health status, degree of disease, and physical capacity, but there is considerable interpersonal variability, as some older adults enjoy very good health and others reveal fast-appearing weakness, disability, and frailty. Among the individual factors which have been studied, self-efficacy is considered the most consistent predictor for beginning to practice physical activity and keeping it up in the long term [7].

Other aspects to bear in mind are that older adults walk slower, have less muscular strength, a worse memory and reasoning capacity, and respond slower to cognitive tasks than younger adults or their younger selves [1]. The sedentary lifestyle which predominates in the elderly provokes the premature appearance of health problems, illnesses, and frailty. Tests show that regular physical activity is safe for healthy and frail older adults and that the risk of developing cardiovascular and metabolic diseases, obesity, falls, cognitive deterioration, osteoporosis, and muscle weakness diminishes if they regularly engage in activities that can range from low-intensity walking to more vigorous sports and resistance exercises [7].

Older adults have shown a decrease in their physical activity levels and self-perceived health status over time, with higher indices of illness and more difficulties in walking and going up stairs [1,6].

As stated by Boulares et al. [8], the aging process usually leads to a decrease in cognitive function, deterioration of the sensory structure, and weakening of the musculoskeletal system. This impacts postural control during static and dynamic activities like walking, which increases the risk of falls in older adults.

**Figure 1:**
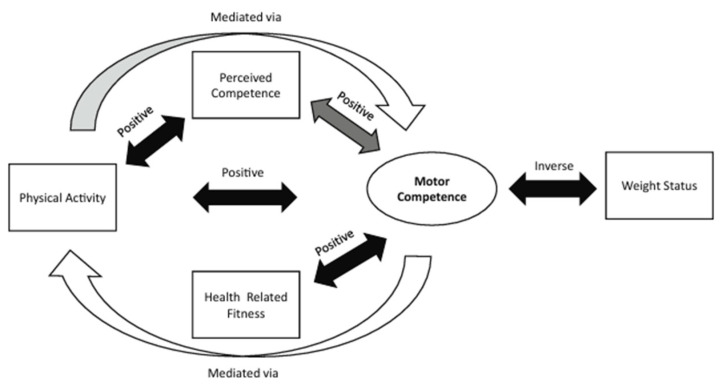
Research consensus on motor competence and health-related variables. The black arrow indicates extensively tested to have a consistent relationship; the dark gray arrow indicates moderately tested to have a variable relationship; the partial gray arrow indicates partially tested, with some evidence; finally, the white arrow indicates limited testing. The direction of the relationship is indicated above the arrows [9].

We should consider that current evidence indicates that motor competence is positively associated with perceived competence (referring to an individual’s perception of their actual movement capabilities) and many aspects of health, including physical activity and physical aptitude [9]. Figure 1 shows the relationships between health-related fitness, perceived competence, physical activity, and the risk of obesity. The authors based their study on previous investigations whose hypothesis was that the lack of development of motor competence leads to a negative spiral of disinterest for physical activity, as children lack the competence and confidence for movement and do not enjoy participating in activities in which they assume they will not be successful. This model could be applied to older people: if because of sedentarism or any other health problem related to age, they lose motor competence and self-confidence, they could enter a vicious circle in which they move even less, thus aggravating these problems.

The results of different studies show that physical activity programs can improve motor and cognitive functions, reducing the risk of falls and improving physical performance, which has a positive impact on motor competence and walking ability [8,10,11].

Knowing the perceived motor competence of older adults is very important due to the protective effect of physical activity and perceived self-efficacy. Programs that combine physical and cognitive exercise can significantly improve neurocognitive performance [12].

In relation to other sociodemographic factors, like social class, it seems that older adults at higher socioeconomic levels are more likely to maintain high levels of physical activity. Those in lower socioeconomic positions are more likely to remain inactive, changing from high levels of physical activity to low levels and from medium levels of physical activity to low levels [13].

Good motor competence in older adults is beneficial for cognitive enhancement, physical fitness, and psychological well-being. These improvements contribute to better daily functioning, reduced fall risk, and enhanced quality of life, underscoring the importance of incorporating motor–cognitive training into regular activities for older adults [14,15,16].

Motor competence in older adults is closely linked to their perception of health, their walking ability and their participation in physical activity, so maintaining motor competence is a priority in these age groups. Understanding these relationships can help in the adaptation of interventions to improve quality of life in older populations.

For all of these reasons, it is necessary to take the environment into account in order to establish proposals for action to encourage the practice of sports, and consequently the motor competence perceived by older adults, to improve the current negative effects of sedentary lifestyles on public health [17].

Therefore, the main purpose of this study was to analyze older adults’ perceived motor competence. The specific purposes were to analyze if sociodemographic characteristics (sex, age, and social class), satisfaction with health status, the type of demand for physical activity and sport, and walking habit influence subjects’ perceived motor competence.

## 2. Materials and Methods

### 2.1. Participants and Study Design

This research followed a quantitative descriptive methodology using a survey. A total random sample of 933 individuals aged 65 years and older (74.12 ± 6.55) that were legal residents in Spain (men = 438; women = 495), with this being the only criterion for inclusion, participated in this cross-sectional study conducted via face-to-face interviews using structured questionnaires.

According to the municipal electoral roll, this population, aged 65 or over, consisted of 7,484,392 people, with men comprising 42.28% of the total and women 57.72%. For sample calculation, working with a 95.5% confidence interval, a ±3.33% permitted sampling margin of error, and supposing that the least favorable case of *p* in population variance equaled 50—hence, q = 50—the sample size needed to be 902 elderly people [18]. However, initially, more interviews were carried out, for a total of 1056 older adults who participated in this investigation. During and after fieldwork, questionnaires were supervised and checked to ensure that they all had been properly completed [19,20]. A total of 123 questionnaires were deleted because they were not completed correctly, so the final sample comprised 933 subjects.

The selection of the sample was probabilistic, clustered, and multi-phased [21]. In this type of samples, the selection of units was carried out in different stages. In stage I, eight municipalities were chosen according to the following different demographic sizes: less than 10,000; between 10,001 and 50,000; between 50,001 and 100,000; and more than 100,001 inhabitants. In stage II, the specific areas in which the questionnaire was going to be administered were chosen; this was carried out in a simple, random way. In stage III, the actual streets were selected for the survey. In stages IV, V, and VI, each interviewer selected the particular surveyed hallway, floor, and entrance gate to the house in which the questionnaire was going to be used; this was established from a route chosen by random sampling. Finally, the last stage was the conducting of the survey with the elderly people recruited.

The interviewers were chosen and trained in the administration of the standard research questionnaire, were explained the routes or itineraries that had to be followed to contact potential interviewees, and the selection criteria to choose the elderly subjects to be interviewed (to be 65 or over and a resident in Spain). The average time taken to complete the questionnaires was 20 min.

An informative introductory letter was given to all participants, and those who volunteered to accept participated in this study. The questionnaires were administered after obtaining the approval for collaboration from the participants. The main project researcher had to sign a declaration that he would respect all current legislation on human rights, ethics, and biosecurity. In addition, the research was approved by the University Committee and the Spanish Ministry of Education and Science (UPM05-C-11203).

### 2.2. Instrument

The Standardized Questionnaire on Physical Activity and the Elderly [22] was used to measure the demand for physical activity and other related variables in the elderly. This instrument includes 5 dimensions: sociodemographic variables, the type of demand for physical activity, lifecycle variables, socialization agents, and a provision of physical activity variable [22]. Since the variables are categorical, alternative responses are compared using Cramer’s V correlation coefficients [20]. The estimated values ranged from 0.83 to 1.0. According to Cea [20], if the correlation coefficient between the two responses is more than 0.8, questions and indicators are considered reliable. The following variables from the questionnaire were used for the present investigation: sociodemographic variables, the type of demand for physical activity, motor competence, walking habit, and self-perceived health.

Regarding the type of demand for physical activity, there were three categories:

- Established demand was formed by those people who practiced one or more physical activities or sports;
- Latent demand was formed by those people who did not practice due to some obstacle or barrier but who were interested in practicing one or more physical activities or sports;
- Absent demand was formed by those people who did not practice and who were not interested in practicing any physical activities or sports.

An example of each category is shown below:

- Do you go for a walk or a stroll? Yes or no.
- Are you satisfied with your state of health? Not–somewhat, not–quite, not–very, somewhat–quite, somewhat–very, or quite–very.
- To which social class do you belong? Medium/low, medium/medium, or high/medium.
- Not including walking, do you perform any sporting activity or physical exercise during the week? Yes or no. If no, would you like to perform any sporting activity or physical exercise during the week? Yes or no.

To carry out the data analysis of the motor competence variable, only the items related to motor competence were selected, so on a scale of 0 to 6, 0 was considered a very poor perception and 6 a very good perception of motor competence by the elderly.

In terms of age, for the analysis of the results, the older adults were grouped into two categories: 65 to 74 years old and 75 years old and older.

### 2.3. Statistical Analysis

All statistical analyses were performed using the SPSS software v.29.0 (IBM, Chicago, IL, USA). The normality of each variable was initially tested with the Kolmogorov–Smirnov test. Since all the variables did not follow a normal distribution (*p* < 0.05), they were analyzed with non-parametric statistics. Descriptive data were presented as percentages, median, and percentiles. The Mann–Whitney U test and the Kruskal–Wallis test were performed to analyze the differences between groups in the motor competence and other variables; pairwise comparisons were performed. The significance level was set to *p* < 0.05. The effect sizes of the Mann–Whitney U test were expressed with Rosenthal’s r (r) with values of 0.1, 0.3, and 0.5 for small, medium, and large effects, respectively. The effect sizes of the Kruskal–Wallis test were expressed with epsilon squared (ϵ^2^), with values of 0.01, 0.06, and 0.14 for small, medium, and large effects, respectively.

## 3. Results

Table 1 shows that the younger group of subjects (≤74 years) perceived their motor competence more satisfactorily (Mdn = 3) than the older subjects (≥75 years) (Mdn = 2), revealing a statistically significant relationship between both variables (*p* < 0.001; r = 0.23). With regard to sex, the men were the ones who considered themselves to have greater motor competence compared to the women (men: Mdn = 3; women: Mdn = 2), revealing a statistically significant relationship between both variables (*p* < 0.001; r = 0.16).

**Figure 2:**
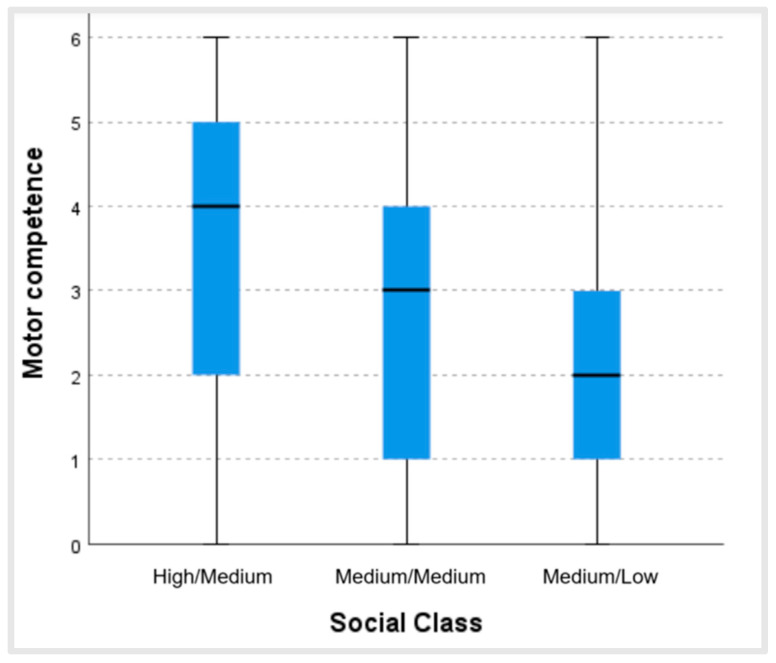
Values of perceived motor competence for each level of social class.

Figure 2 shows that the higher the social class, the better the perceived motor competence. Moreover, there were statistically significant differences between the social class of the subjects and their perceived motor competence (*p <* 0.001; ϵ^2^ = 0.05). These differences existed between all the groups analyzed: medium/low–medium/medium (test statistic 89.89; dev. error. 18.55; *p <* 0.001); medium/low–high/medium (test statistic 198.73; dev. error. 32.32; *p <* 0.001); and medium/medium–high/medium (test statistic 108.84; dev. error. 31.16; *p =* 0.001). 

**Figure 3:**
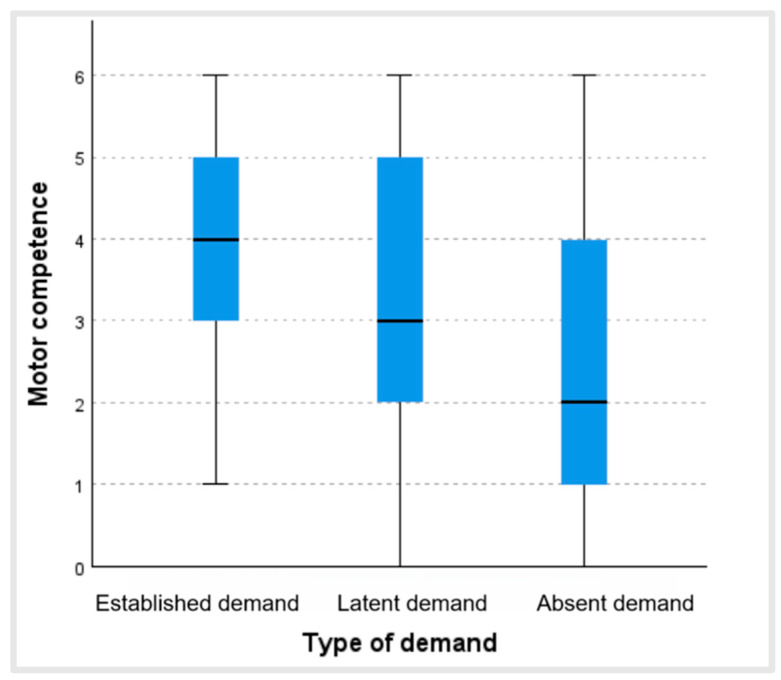
Values of perceived motor competence according to the type of demand.

Figure 3 shows that people who regularly practiced physical activity and/or sport (established demand) were the ones who had a better perception of their motor competence (Mdn = 4), followed by those who did not but would have liked to practice physical activity and/or sport (latent demand) (Mdn = 3), and, lastly, older adults who did not practice physical activity and/or sport and had no desire to start (absent demand) (Mdn = 2). Moreover, there were statistically significant differences between the level of physical activity and/or sport practiced and perceived motor competence (*p <* 0.001; ϵ^2^ = 0.13). These differences were revealed between all the groups: absent demand and latent demand (test statistic 154.69; dev. error. 26.55; *p <* 0.001); absent demand and established demand (test statistic 240.49; dev. error. 23.38; *p <* 0.001); and latent demand and established demand (test statistic 85.80; dev. error. 32.15; *p* = 0.023).

**Figure 4:**
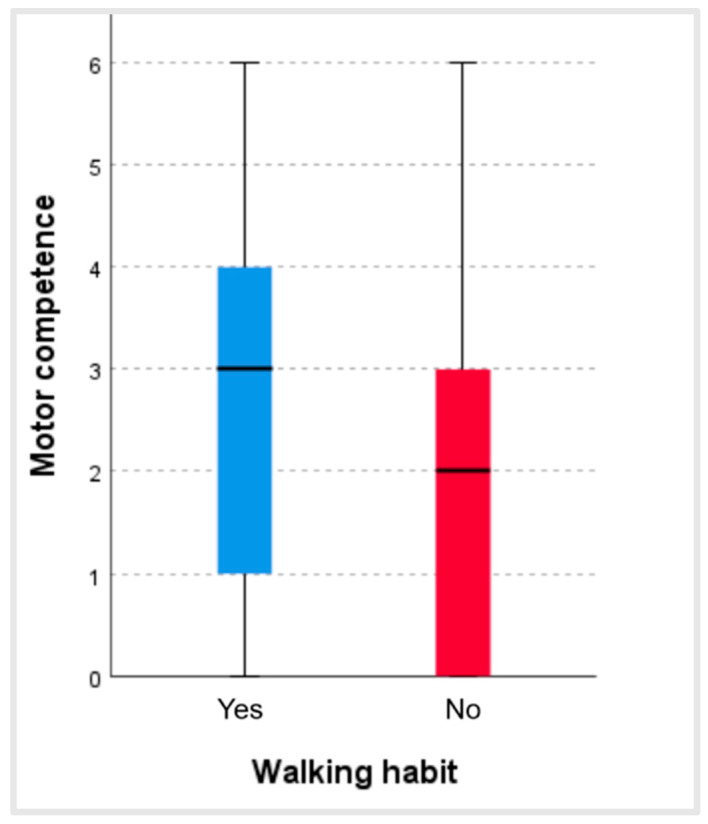
Values of motor competence according to the walking habit.

With regard to the walking habit, there was a statistically significant relationship between habitually walking and perceived motor competence (*p <* 0.001; r = 0.14). Figure 4 shows that the people who stated that they usually walked obtained higher scores in perceived motor competence (Mdn = 3) than those who did not usually walk (Mdn = 2).

**Figure 5:**
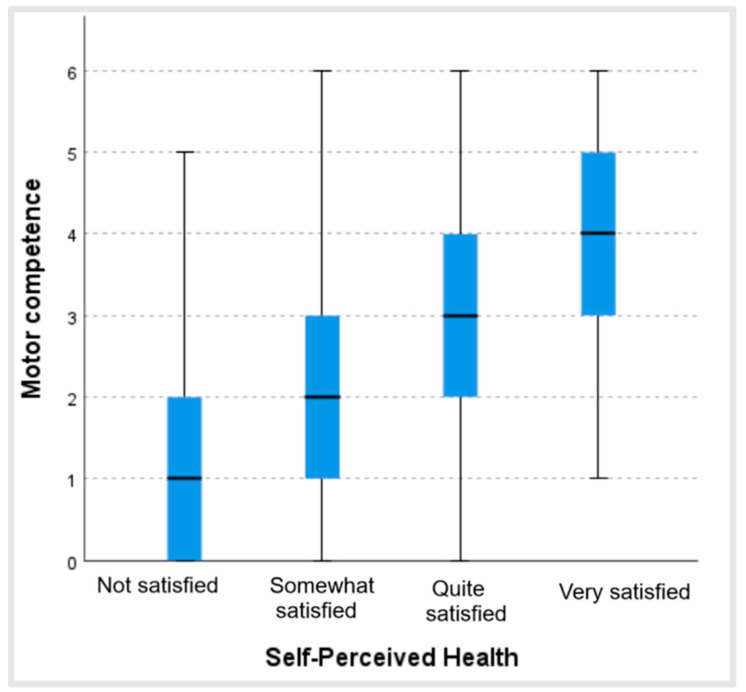
Values of perceived motor competence according to satisfaction with health status.

Figure 5 shows that the more satisfied people were with their health status, the higher their score in perceived motor competence, revealing a statistically significant relationship between both variables (*p* < 0.001; ϵ^2^ = 0.21). These significant differences existed between all the groups analyzed: those who were not satisfied and those who were somewhat satisfied with their health status (*p* = 0.002); those who were not satisfied and those who were quite satisfied (*p <* 0.001); those who were not satisfied and those who were very satisfied (*p <* 0.001); those who were somewhat satisfied and those who were quite satisfied (*p <* 0.001); those who were somewhat satisfied and those who were very satisfied (*p <* 0.001); and those who were quite satisfied and those who were very satisfied (*p <* 0.001) (Table 2).

## 4. Discussion

The purpose of the present research was to analyze if sociodemographic characteristics, satisfaction with health status, type of demand for physical activity and sport, and the walking habit influenced the perceived motor competence of the subjects.

The results showed that the people who were included in the “established demand” group, those who practiced physical activity, were the subjects who had the best perception of their motor competence in both age groups, scoring above the older adults in the “latent demand” group (who desired to practice physical activity but were confronted with barriers which prevented them from this).

Moreover, the data showed that there was a statistically significant relationship between habitual walking and perceived motor competence. In the study by Bastian et al., carried out with older adults with multiple morbidities, the authors concluded that the qualitative aspects of walking, especially during changes in direction, were the main determinants of the quantitative dimensions of physical activity (duration, frequency, and intensity) [23]. For this reason, although in most cases walking may be an insufficient activity, it is one which should be taken into account as a complement to physical activity, both in older adults who present greater motor competence and in those who have a lesser degree of competence. Some research shows that walking performance in older adults is a strong biomarker of health [24,25].

It is evident, as stated by Yin et al. [26], that motor function is essential for the independence of older adults, but they also affirm that, unfortunately, frail individuals who have difficulty walking or standing have limited access to physical activity programs and assessments of motor function.

The results obtained in the present study confirm those found by authors like McPhee et al. [7], Boulares et al. [8], Schättin et al. [10], Wollesen et al. [27], and Zwingmannet al. [12], researchers who indicated that the practice of physical activity could be a protective factor against the aspects which cause the deterioration associated with aging, improving motor and cognitive functions, reducing the risk of falls, and enhancing physical performance, which brings about better motor competence and walking capacity. In this same vein, Whaley and Schrider [4] affirm that adherence to exercise in older adults is influenced by their self-perception of physical competence.

In the systematic review by Levin et al. [11], it was found that physical activity can mitigate or even improve the motor and cognitive abilities of older adults. Their review centered on studies which examined the dual effects of different types of physical training (like balance training, aerobics, strength, group sports, etc.) in motor and cognitive tasks in older adults without known disabilities. Among their main conclusions, they indicate that physical exercise can improve mood, relieve anxiety and depression, and enhance general cognitive functions like memory, attention, inhibition, and speed of processing, as well as mobility, balance, and fine motor control of the upper limbs.

The older adults who did not practice physical activity nor desired to start (absent demand) were those who had the worst perception of their motor competence. It should be borne in mind that a worrying percentage of older adults do not practice physical activity. Abel et al. [23] evidenced highly inactive behaviors in older adults with cognitive deterioration (89.6% inactivity) and a high incidence of walking deficits in a study carried out with 94 older adults. Taking into account the total number of older adults in Spain, in our investigation, 69.9% of the total made up the absent demand group. More concerning is the confirmation provided in the study by Latorre-Román [1] of the tendency for the prevalence of the practice of physical activity to diminish. However, the trend towards diminishing physical activity practice over time found in this study does not support prior investigations carried out in developed countries which indicated that the levels of physical activity seemed to be increasing [28]. New research is necessary on this topic to clarify this tendency.

Analyzing the results of the present investigation, it can be seen that the people in the younger age group reveal greater perceived motor competence than the older group. This could be due to the fact that, in spite of the enormous interpersonal differences, the increase in chronological age generally leads to more functional deterioration and the appearance of physical health, cognitive, and, on occasions, social problems [1,6,7,8]

According to gender, men reported better motor competence than women. As stated earlier, Whaley and Schrider [4] highlight the importance of perceived motor competence for motivation and adherence to exercise, and these authors affirm that women tend to be more critical of their body image, which may influence their motivation to participate in physical activities. In the study by Latorre-Román et al. [1], the results reveal that women experience a greater degree of illness or health problems than men and greater difficulty in walking and going up and down stairs. With regard to the research by Fastame et al. [29], it was shown that, although men achieved a better performance in certain parameters of mobility (which the authors attribute to the intrinsic anthropometric differences between men and women), both sexes could benefit equally from maintaining motor competence in terms of their perceived health status.

In relation to the sociodemographic variable of social class, in our study, it could be observed that the higher the social class of the older adults, the better their perceived motor competence. These data point in the same direction as previous research indicating that older adults in higher sociodemographic strata are more likely to maintain high levels of physical activity than those in lower socioeconomic strata, who are more likely to remain inactive and change from high levels of physical activity to low levels or from medium levels to low levels [13]. It may be that those with more resources have more advantages for participating in physical activity and sports programs and, moreover, have access to more information, becoming aware of the benefits and importance of including physical activity in their daily routine. However, this is just a hypothesis and needs to be investigated to delve more deeply into these variables.

With respect to perceived health, the subjects who were more satisfied with their health status obtained higher scores in perceived motor competence. This relationship could be explained considering that enjoying better health could be motivating for older adults in relation to their practice of physical activity, which, in turn, could lead to better motor competence. Moreover, being a physically active person, as stated earlier, is related with better perceived motor competence, which could have influenced the older adults in this study to indicate a better perception of their health status. Whaley and Schrider [4], in their research, aimed to discover the reasons why older adults practiced physical exercise, and among their main concerns were maintaining or improving their physical capacity, health, and functional independence.

In this regard, Robinson et al. [9] explain that motor competence is positively associated with several health-related aspects like physical activity, cardiorespiratory capacity, muscular strength, and maintenance of a healthy weight, aspects which suggest that greater motor competence could lead to better results for one’s health [9,12]. In this same vein, Fastame et al. [29] state that there is a significant relationship between motor competence and self-perceived psychological well-being, as older adults with better motor competence tend to report higher levels of emotional well-being, which suggests that maintaining motor skills is essential for perceived health status.

Among the limitations of this study, we can cite that it was a cross-sectional and purely descriptive investigation, which prevented us from diving more deeply into the causes of why the older adults in our sample perceived their motor competence as described or from implementing intervention strategies to try to improve their perception of this variable. This study did not address potential confounding factors, such as pre-existing health conditions, medication use, or psychological factors influencing motor competence perception, because the sample had to be a representative sample of the reality under study. In addition, the limitations of self-reported measures must be taken into account.

Regarding future lines of research, the above aspects could be taken into account to control possible confounding factors. In addition, this study could be replicated in other countries to see the reality of the variables analyzed at the international level. In view of this, it should be considered that there may be other factors related to the context of each country that could influence the results. Another possible line of research could aim to delve more deeply into the barriers that prevent older adults from practicing physical activity in the latent demand group and discover how these possible barriers relate to motor competence. Moreover, based on the results obtained, physical activity programs could be implemented with measures focused on the population with a lower level of motor competence and perceived health to reduce sedentarism in these groups and increase the quality of life of these older adults. For practical purposes, measures could be implemented such as offering free or low-cost programs tailored to the interests of older adults for physical activity, as well as taking into account aspects such as the proximity of sports facilities to their residence or providing means of transportation to attend the activities.

## 5. Conclusions

Practicing physical activity is a crucial factor for older adults. In our study, the people who reported higher levels of physical–sports activity and those who declared that they habitually walked presented better perceived motor competence, which is an essential aspect for adherence to physical activity programs. Moreover, the people in the younger group, those from a higher socioeconomic level, and men had greater perceived motor competence compared to the other groups, organized in terms of sociodemographic variables.

Finally, there appeared to be a statistically significant relationship between the variable of perceived health status and perceived motor competence in older adults.

The results of the present research may serve as a guide to people and institutions responsible for fomenting physical activity and sports programs for older adults. On the one hand, it confirms the results reported in other investigations on the need for this population group to practice physical activity. On the other, it could serve as a basis for implementing strategies aimed at those groups who present lower perceived motor competence, with the purpose of reducing sedentarism and improving their quality of life.

## Figures and Tables

**Table 1:** The Mann–Whitney U test for age and sex in relation to perceived motor competence. Mdn = Median.

Variables		N	Mdn	Sig.
Age	≤74	510	3	0.000
	≤75	423	2	
Sex	Female	495	2	0.000
	Male	438	3	

**Table 2:** Pairwise comparisons of satisfaction and health status.

Sample 1–Sample 2	Test Statistic	Dev. Error	Test Statistic Dev.	Sig.	Adjusted Sig. ^a^
Not–Somewhat	−114.633	31.390	−3.652	0.000	0.002
Not–Quite	−270.262	28.919	−9.346	0.000	0.000
Not–Very	−390.712	32.360	−12.074	0.000	0.000
Somewhat–Quite	−155.629	21.989	−7.077	0.000	0.000
Somewhat–Very	−276.078	26.352	−10.477	0.000	0.000
Quite–Very	−120.450	23.354	−5.158	0.000	0.000

Each row proves the null hypothesis that the distributions of sample 1 and sample 2 are equal. Asymptotic significance (two-tailed tests) is shown. The level of significance is 0.5. ^a^ The significance values have been adjusted with the Bonferroni correction for several tests.

## Data Availability

Data supporting the reported results can be found at the following link: https://drive.google.com/drive/folders/1tuxIzhrzoYHxPzwM4UrqO6vzXXw2isnk?usp=sharing (accessed on 8 April 2025).
